# Characterizing Hippocampal Oscillatory Signatures Underlying Seizures in Temporal Lobe Epilepsy

**DOI:** 10.3389/fnbeh.2021.785328

**Published:** 2021-11-25

**Authors:** Thato Mary Mokhothu, Kazumasa Zen Tanaka

**Affiliations:** Okinawa Institute of Science and Technology Graduate University, Okinawa, Japan

**Keywords:** seizures, hippocampus, biomarkers, temporal lobe epilepsy, latent period, pathological oscillations, high-frequency oscillations

## Abstract

Temporal Lobe Epilepsy (TLE) is a neurological condition characterized by focal brain hyperexcitability, resulting in abnormal neuronal discharge and uncontrollable seizures. The hippocampus, with its inherently highly synchronized firing patterns and relatively high excitability, is prone to epileptic seizures, and it is usually the focus of TLE. Researchers have identified hippocampal high-frequency oscillations (HFOs) as a salient feature in people with TLE and animal models of this disease, arising before or at the onset of the epileptic event. To a certain extent, these pathological HFOs have served as a marker and a potential target for seizure attenuation using electrical or optogenetic interventions. However, many questions remain about whether we can reliably distinguish pathological from non-pathological HFOs and whether they can tell us about the development of the disease. While this would be an arduous task to perform in humans, animal models of TLE provide an excellent opportunity to study the characteristics of HFOs in predicting how epilepsy evolves. This minireview will (1) summarize what we know about the oscillatory disruption in TLE, (2) summarize knowledge about oscillatory changes in the latent period and their role in predicting seizures, and (3) propose future studies essential to uncovering potential treatments based on early detection of pathological HFOs.

## Introduction

Temporal Lobe Epilepsy (TLE) is the most commonly medically refractive focal epilepsy (Engel, 2014). It mainly begins in the limbic areas and could be secondarily generalized, resulting in tonic-clonic seizures (Shibley and Smith, 2002; Bone et al., 2012). Studies using electrical recordings and expression of immediate early genes to identify the cellular activations during seizures have found that this secondary generalization plays a role in the increased frequency of seizure episodes in the chronic stage (Kiernan, 2012; Sinel’Nikova et al., 2013) and is one of the risk factors for the phenomenon called sudden unexpected death in epilepsy (SUDEP), one of the leading causes of death for people with epilepsy (Bone et al., 2012). During epileptogenesis, there are numerous changes within the brain: epileptic discharges emerge, altered neural firing patterns due in part to altered neurotransmitter releases, the connection of and communication between cells (Janz et al., 2017; Ren and Curia, 2021), neural death and the aberrant migration of granule cells (Henderson et al., 2014; Cho et al., 2015; Kim and Cho, 2018). In animal models which use chemoconvulsants for induction, these disease features are elicited similarly to the human condition (Curia et al., 2014; Paschen et al., 2020).

The hippocampus is one of the main foci of TLE and, simultaneously, a key area involved in various types of memory, including contextual memory and spatial navigation. Therefore, its pathology can lead to many cognitive deficits, including learning and memory (Andersen, 2007). The above features, which facilitate TLE progression, also correlate to higher cases of cognitive decline in both humans and animal models (Cho et al., 2015).

The distinctive three phases of TLE are (1) Acute phase: in which there is a prolonged seizure due to a brain insult, (2) Latent phase: is a period between the initial precipitating (IPI) and the chronic stage without any exhibition of behavioral seizures, and (3) The chronic phase: is marked by spontaneous recurrent seizures (SRS) (Panayiotopoulos, 2005; Curia et al., 2008; Kandratavicius et al., 2014; Janz et al., 2017). These are seemingly unprovoked seizures in the people with epilepsy, which occur weeks, months, or even years after the first incident (Maguire, 2016). In human cases, epilepsy is usually diagnosed at the chronic state, although the consensus is that many neurological changes occur long before the start of observable recurrent seizures (Cho et al., 2015; Maguire, 2016). The IPI itself is traced back using self and caretaker reports during consultation. In modern studies, animal models of TLE can distinctly depict these three stages, making them good models for studying TLE.

Sir William Gowers first described these stages and have been kept as a standard definition throughout the decades (Gowers, 1901). However, the latent period is the least understood. There is a high variability of its duration (making it challenging to define temporally), and there is no typical behavioral output (making it difficult to detect). For example, if seizures begin after 3 days after an IPI, we may be better able to link the two events, but there is a question about whether this can be regarded as latency at all vs. a series of seizures occurring 6 years after an IPI, in which a precise latency can be defined, but the details of the IPI may be less accurate due to time lapse (Löscher et al., 2015). The question about what latency constitutes and its role in developing TLE has been open for a long time. It is pressing even more now that we have better tools to assess the mechanisms underlying progression (Cai et al., 2021). Insights about brain activity during the so-called “latent period” may lead to better targeting procedures for interventions in TLE, possibly through earlier therapeutic windows.

## The Latency Controversy

There is currently an exciting debate in the epilepsy field: One school of thought is that the IPI presents all the necessary features to elicit SRS, and even without a latent period, the chronic seizures will begin (Rattka et al., 2011; Löscher et al., 2015). This view is based on observing varied results from some human and animal studies in which some seizures began almost immediately after the IPI (Löscher et al., 2015). Another thought is that latency is a prerequisite process in epileptogenesis during which changes occurring in the brain, including circuit rewiring, will culminate in the seizures elicited in chronic periods (Panayiotopoulos, 2005; Lee et al., 2017). This is based on a traditional view of the stages of epilepsy, whose central gap is the lack of explicit knowledge about brain activity in the absence of seizures. Maguire (2016) suggests that many cellular and molecular changes occur in the brain, making the emergence of SRSs more likely. It is now appreciated that dubbing the latent period as the “silent period” may be misleading: While behaviorally, there are no seizures, the brain activity may show a different picture. At this juncture, the presence of a latent period seems more likely as it is reported in more cases than not (Lévesque and Avoli, 2013), but what is still unclear is what happens during this time. To answer that, we need to evaluate differences between a latent epileptic brain and a healthy brain, and one of the ways to characterize neural population activity is through brain oscillations.

## Non-Pathological Oscillations

*Brain oscillations* are local field potentials generated by microcircuits of specific cell types due to internal influences or external or cognitive demands (Menendez de la Prida and Huberfeld, 2019). Hippocampal theta oscillations (4–12 Hz) are very prominent in rodents, typically observed when the animal attends to a novel object or when it is paying attention or performing a task (Başar and Güntekin, 2008; Belchior et al., 2014), although their expression and characterization in human studies are still controversial (please see Herweg et al., 2020). These oscillations have been found persistently in the CA1, CA3, and DG of rodents during voluntary, preparatory, or exploratory movements, as well as during rapid eye movement (REM) sleep (Buzsáki, 2002), associated with a replay of memories and dreaming (Louie and Wilson, 2001). For example, during a contextual fear conditioning task, the theta oscillations were observed in both the hippocampus CA1 and the lateral amygdala, and their synchronization increased during retrieval of fear memories (Seidenbecher et al., 2003). Researchers further found that synchronization of neuronal activity in addition to theta bursts is correlated with both learning and retrieval of contextual fear memory (Zhou et al., 2020). Potentiation is most efficient during theta band oscillation: In 2009, a study showed that low-frequency stimulation on the perforant pathway induced theta rhythms in the dentate gyrus, which increased EPSPs and Ca^2 +^ influx (Tsanov and Manahan-Vaughan, 2009). Theta oscillations are therefore conducive to synaptic plasticity. The same has been found in other regions, such as the medial prefrontal cortex (mPFC), where learning depends on theta oscillations. The theta-dependant neuronal synchrony is said to underlie memory transfer (Paz et al., 2008).

Gamma oscillations are high-frequency oscillations (30–80 Hz) observed in many brain parts, including the hippocampus (Buzsáki and Schomburg, 2015). Gamma oscillations have brief durations and arise from the interplay of excitation and inhibition in local cell assemblies, with the drive from PV^+^ inhibitory neurons playing an essential role (Buzsáki and Wang, 2012).

Gamma oscillations are frequently observed alongside theta waves. Theta-gamma coupling is highly regulated, and any disturbances in this system may lead to abnormalities and pathology in the brain (Zhang et al., 2016).

In the hippocampus, another type of physiological oscillation is called sharp waves (SPW). They are high amplitude, low-frequency patterns in the LFPs (Sullivan et al., 2011). They are often accompanied by a higher frequency of activity (ripple), and this phenomenon, where both waveforms co-occur (sharp-wave ripples, SWRs), is observed in various animals, including humans (Buzsáki, 2015). It tends to dominate the brain during the awake phase but immobile moments or non-REM sleep and has been linked to processes that support memory consolidation (Sullivan et al., 2011; Buzsáki, 2015; Oliva et al., 2018). In a study using a spatial discrimination task in rats, it was found that trained rats had a more sustained sharp-wave ripple activity following the task and during the non-REM stage of their sleep. Compared to the non-trained rats, the experimental group showed an increase in ripple density with increasing performance accuracy (Ramadan et al., 2009). During these SWRs in non-REM sleep, neurons active during a task are re-activated with the identical spike sequences but in a shorter timescale replay (Wilson and McNaughton, 1994; Skaggs and McNaughton, 1996; Lee and Wilson, 2002). Moreover, selective interruption of SWRs during awake causes a deficit in spatial learning (Jadhav et al., 2012). Because of these properties, the SWR was then considered the neural substrate of memory consolidation (Wilson and McNaughton, 1994; Ramadan et al., 2009; Buzsáki, 2015).

Oscillations whose spectral power goes beyond the limits of the gamma band are known as high-frequency oscillations (HFOs) (125–250 Hz) and very high-frequency oscillations (vHFOs) (250–500 Hz) (Kucewicz et al., 2014). In other studies, the former are referred to as ripples and the latter as fast ripples (Bragin et al., 1999). In a study on the visual cortex in humans, a visual HFS paradigm enhanced plasticity in the visual cortex by raising the phase synchrony of theta oscillations (Hamilton et al., 2020). These results imply that HFOs too facilitate neural plasticity and network rewiring in healthy animals for learning and memory. However, aberrant oscillations may lead to aberrant types of plasticity: for instance, the higher the HFO frequencies, the higher the likelihood of a disease, specifically above 300 Hz, the proposed physiological oscillatory frequency boundary (Pail et al., 2020). Therefore, we need to examine changes leading to pathological oscillations carefully.

## Pathological Oscillations

**FIGURE 1:**
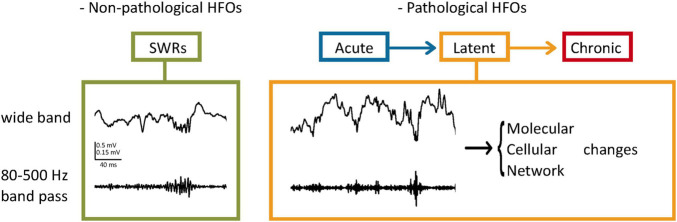
Pathological HFOs and their possible role during the latent phase of TLE. **(Left)** SWRs as an example of non-pathological HFOs. In the hippocampus, HFOs (80–200 Hz) called ripples are associated with high amplitude activities (Sharp Wave Ripples, SWRs) and contribute to neuronal plasticity and memory consolidation. **(Right)** Pathological HFOs during the latent phase of TLE. Higher frequencies of oscillations (200–500 Hz) are observed between the acute and chronic stages without behavioral expression of seizures. Neuronal activities leading to the pathological HFOs are suggested to result in long-term molecular and cellular changes. Circuit rewiring caused by these changes might make the more brain areas prone to SRSs, leading to further progression of TLE.

Pathological oscillations are fast oscillations occurring at higher frequencies than the average observed in healthy brains (Engel et al., 2009; Ewell et al., 2019). Bragin et al. (1999) observed that in normal and in kindled rat brains, field recordings did not exceed 200 Hz, while in the kainate acid (KA)-treated animals, recordings of > 200 Hz were observed near the injection site, which was also the sclerotic tissue. Their study characterizing ripples and fast ripples defined *ripples* as oscillations occurring at 100–200 Hz and lasting 50–150 ms, while *fast ripples* were the 200–500 Hz oscillations lasting 10–100 ms (Bragin et al., 1999). Although sometimes frequency bands of pathological oscillations may overlap with physiological oscillations (Ibarz et al., 2010; Pail et al., 2020), one study suggests that the peak amplitudes of pathological oscillations are highly variable compared to the more consistent physiological recordings (Ewell et al., 2019). Apart from features of the HFOs, the locations in which they occur are also of importance. For instance, according to Engel et al. (2009), oscillations occurring at 250–600 Hz in the hippocampus would be considered pathological, while in the neocortex, they may be facilitating physiological processes. Additionally, pathologic oscillations seem to occur regardless of the brain state, while physiological HFOs are linked closely with specific tasks (Ewell et al., 2019). Buzsáki (2015) theorized that the threshold of conversion from an HFO to a pathological HFO is minimal, therefore in highly excitable circuits such as the hippocampus, a perturbation of normal HFO is likely to lead to seizure disorders. The appearance of these HFOs during the latent and, more frequently during chronicity implies that a progressive change in the network generates this activity (Figure 1; Assenza et al., 2020).

In a Ca^2+^ imaging study, Lillis et al. (2015) found an increase in local hippocampal synchronization stemming from the recruitment of more cells from the early stages following induction of epilepsy. In the chronic phase, there are more HFOs than in the healthy or the early seizure stages, and more than in the acutely epileptic brain, suggesting a progressive series of changes such as network reorganization throughout TLE development that could lead to long-term pathophysiology (Lévesque et al., 2018). Another finding following seizure induction was that the earlier the HFOs were exhibited during latency, the shorter the latency (Jiruska et al., 2017). This relationship could be one of the explanations for why some latencies are shorter than others. It also leads us to the natural assumption that perhaps the latent period represents the rewiring of the brain and that there is a threshold for these aberrant activities, which, when met, begins the chronic stage of TLE. When the latent period transitions into the chronic phase, there is also a change in the site of HFO generation, from the hippocampus to the entorhinal cortex (Jiruska et al., 2017), and an increased duration of the HFOs near the beginning of SRS (Lévesque et al., 2018). The dynamics and presentation of HFOs, once understood, can be used to detect TLE stages and serve as a warning before the looming first SRS.

Song and colleagues observed stronger temporal lobe coherence during resting states in people with chronic epilepsy versus healthy controls (Song et al., 2013). The theta, gamma, and beta power are heightened during the ictal part of the seizure, while delta power is high in the interictal stages (Sharma et al., 2018). A study in the early 90 s showed a correlation between theta oscillations and the attenuation of seizures. Stimulating the medial septum at 4–8 Hz after a pentylenetetrazole-induced seizure stopped the ongoing seizures within seconds, while the lesioning of the medial septum removed the theta waves and heightened susceptibility to and frequency of seizures (Miller et al., 1994), further emphasizing the theta-SWR dichotomy even in pathology. Recent evidence supports this claim through experiments using continuous deep brain stimulation on the medial septum in animal models of TLE. The results were significant reductions in seizure severity, the incidence of generalized seizures and SE, and the total number of generalized seizures. The 5 Hz stimulation yielded the most significant effects on all measurements (Wang et al., 2021). A slice preparation experiment showed that directly stimulating the GABA-ergic neurons in the medial septum (MS) achieves the same effect (Hristova et al., 2021). The results coincide with the already established role of the MS that is the generation and input of theta oscillations to the hippocampus (Buzsáki, 2002; Watson et al., 2019).

High-frequency oscillations may also be a crucial seizure-prediction tool that shows the locations of seizure generation and the time window to the subsequent seizure, and possibly, they could define the stage of the epileptogenesis (Ibarz et al., 2010). In a human study using intracranial EEG and HFO detection systems, machine learning algorithms could reliably recognize patterns of brain activity that preceded the seizures up to 30 min and discern them from non-seizure-related activity (Scott et al., 2021). Due to higher HFO instances in the ipsilateral hippocampus rather than the contralateral side can be used as a biomarker for the seizure focus (Řehulka et al., 2019) path. Non-invasive tools have already been developed for better detection with minimal discomfort for people with TLE (Cai et al., 2021). As such, not only can HFOs be used to predict the first spontaneous seizure (after latency), but they could also be used to predict the ongoing seizures throughout chronicity and their site of generation (Table 1 summarizes the important studies which observed pathological HFOs either in the human and rodents). Therefore, we can appreciate the potential utilization of HFOs in the clinical setting for people with either acute and chronic epilepsy.

**TABLE 1:** Studies evaluating pathological HFOs (pHFOs) in the epileptogenic regions.

**Study**	**Subjects**	**Recording location**	**Detection parameters**	**Findings/Conclusion**
Bragin et al., 1999	Rats (KA), Humans	Hippocampus (CA1, DG), Entorhinal cortex	Depth electrodes, microelectrodes	Fast ripples (250–500 Hz) were only found in the hippocampus of KA-treated rats and epileptic humans; fast ripples are defined as pathologic.
Staba et al., 2002	Humans	Hippocampus, Entorhinal cortex	Depth electrodes	More HFOs were detected in the hippocampal sites ipsilateral to onset location compared to contralateral sites.
Worrell et al., 2004	Humans	Temporal, frontal lobe	Video-EEG during sleep and wakefulness	pHFOs found in seizure onset zones and could predict onset within a specific timeframe in neocortical epilepsy.
Burnos et al., 2014	Humans	Various: Amygdala; Entorhinal cortex; Frontal anterior; Frontal lobe; Frontal posterior; Hippocampus; Mesial temporal lobe; Perirhinal cortex; Temporal basal anterior; Temporal basal posterior; Temporal depth frontal; Temporal depth lateral	iEEG	Location of pHFOs overlapped with the location of onset.
Řehulka et al., 2019	Humans	Hippocampus, amygdala	Depth electrodes	Ripples were more prominent in epileptic rather than non-epileptic brains. Higher frequencies were observed near sclerotic tissue.
Scott et al., 2021	Humans	Temporal, frontal lobe	iEEG	Pre-ictal and inter-ictal HFOs can in some cases be differentiated, and changes in the frequency of the HFOs can be used as predictors of seizures.
Jiruska et al., 2010	Rats (low-calcium ACSF perfusion of slices)	Hippocampus (CA1)	Glass pipettes, extracellular field potentials	HFOs build up before the seizure onset.
Ibarz et al., 2010	Rats (KA), Computer simulations	Hippocampus (DG, CA1, CA3), computational models	Silicon probes, computational models	Populations spikes and emergent spikes cause fast ripples, region-specific differences in synchronicity during HFOs.
Salami et al., 2014	Rats (Pilocarpine)	Hippocampus (CA1), Entorhinal cortex	Depth electrodes	pHFO dynamics and distribution changes during latency and chronic stage, inter-ictal spikes change before and after the first spontaneous seizure.
Jones et al., 2015	Mice (KA)	Hippocampus (CA1, DG)	Glass pipettes and silicon probes	pHFOs begin in the early latent period in CA1, and the majority are observed in DG. Peak amplitudes increase with epileptogenesis.
Ewell et al., 2019	Rats (KA)	Hippocampus (CA1)	LFP and single-unit recordings with tetrodes	pHFOs found only in epileptic brains independent of brain state and are associated with inter-ictal spikes.

## Cellular Underpinnings of High-Frequency Oscillations

The cellular mechanisms underlying the generation of pathological HFOs are still unclear, and more work is needed to understand them. There are, however, some theories that have been suggested to understand how HFOs arise. For example, some research states that the generation of pathological HFOs during seizures is due to the synchronous firing of pyramidal cells and the relative reduction of activity from the interneurons (Pail et al., 2020).

Jiruska et al. (2017) also posit that the pHFOs represent this fast (order of milliseconds) synchronous firing within aberrantly connected principal cell population (Zijlmans et al., 2012) which is different from normal ripples whose origin is believed to be the activity of interneurons and the summation of their IPSPs in an area (Engel et al., 2009; Pail et al., 2020). Physiological HFOs are said to emanate from the activity of cells in the pyramidal layer of the CA1 and interneurons such as the basket cells, especially PV^+^ cells responding to the bursting of CA3 neurons with a surge of IPSPs (Chrobak and Buzsáki, 1996; Bragin et al., 1999). During an SWR, a physiological oscillation, Ramadan et al. (2009) suggest that the activity of both excitatory and inhibitory neurons in the CA3 is at its peak, but that the firing rate of pyramidal neurons is higher than that of interneurons, causing the synchronous depolarization of cells of the CA1 downstream. From a study utilizing computer simulations, Ibarz and colleagues suggested that both in-phase firing and out-of-phase firing of principal cells in the hippocampus could lead to fast ripples observed in the epileptic brain. Additionally, the degree of synchrony in cellular discharge was found to be much more pronounced in the dentate gyrus than in other parts of the hippocampus, emphasizing that the elicitation of pathologic HFOs is complex and may look different on the single-cell level compared to the population behavior (Ibarz et al., 2010). The pathologic HFOs may also arise due to increased overall excitability caused by interneuron cell death following repeated seizures (Walker, 2015). The demographics of the remaining neurons may impact the molecular characteristics of the region, too, that is, causing a differential release of neurotransmitters or expression of receptors leading to an imbalance in neurotransmission (Menendez de la Prida et al., 2015). Another factor for elicitation of HFOs could be the breaking of the dentate gate. The dentate gate theory states that due to the nature of the granule cells and the local inhibitory circuitry, the dentate gyrus essentially acts as a gate that keeps the probability of seizures low. However, in epilepsy, there seems to be a malfunction in the regulation of this gate, and therefore the granule cells may become overexcitable (Krook-Magnuson et al., 2015). This makes sense when we consider some literature that has found that pathological HFOs are never observed in the dentate gyrus of healthy brains but are a hallmark of those that develop epilepsy (Engel et al., 2009). Importantly, these HFOs are observed in the seizure-generating areas ipsilateral to the site of drug injection and the site of tissue sclerosis within the hippocampus (Krook-Magnuson et al., 2015).

Hippocampal sclerosis may be one of the causes of local network reorganization (Walker, 2015) during the latent period. Since there is substantial neural damage induced from the hypertoxicity during the seizure development, the affected tissues endure atrophy and gliosis (Curia et al., 2008; Walker, 2015) throughout latency (Kim and Cho, 2018). A second cause may be the presence of HFOs within the latent period, which intensifies as the chronic stage nears. HFOs have been shown to facilitate synaptic changes in healthy neurons; however, if the HFOs occur more frequently and in different regions (Lévesque et al., 2018) than in healthy brains, this could lead to abnormal synaptic rewiring within the latent period (Janz et al., 2017) which may result in the increased propensity toward seizures. Thus, the relationship between HFOs and the extent of sclerosis in TLE may be another chicken and egg problem.

## Future Directions

Growing evidence suggests that pathological HFOs within the latent period are associated with network rewiring, leading to changes in their expression as epileptogenesis continues. If this is the case, could stopping network rewiring also stop the HFOs? Could stopping the HFOs prevent the TLE development? In 2013, Krook-Magnuson and colleagues developed a method that could detect a seizure onset and optogenetically silence the activity of the hippocampal pyramidal cells in chronic mice. This intervention resulted in a significant reduction in the seizure duration (Krook-Magnuson et al., 2013). One can imagine adapting this approach to detect the HFOs preceding the actual seizure, eliciting inhibition on the seizure focus, effectively preventing the seizure from happening at all.

Since closed-loop devices like the one described above may be invasive, though, inhibition through drugs could offer a less invasive solution (Smith et al., 2016; Vlasov et al., 2018). More specifically, since HFOs typically arise from around the seizure focus, we could investigate the applications of a DREADD mediated inhibition of the cells in the identified area following early detection of HFOs to prevent a seizure.

Löscher et al. (2015) states that many preventative interventions in the form of antiepileptic drugs (AEDs) have not been successful in attenuating chronic seizures. These results could be due to the wrong line of treatment for the type of epilepsy or the non-ideal timing of the therapy. An alternative approach to circumvent that would be to design an experiment for transiently reducing overall neural activity after a seizure rather than targeting a specific circuit to stop the overall network rewiring and aberrant synaptic plasticity changes. Some animals in the wild can achieve this brain state through the process of hibernation, in which neural activity reduces upon temperature reduction (Walker et al., 1977). This long-lasting hypothermic and hypometabolic state was artificially induced in non-hibernating animals using a DREADD mediated activation of hypothalamic Q-neurons (Takahashi et al., 2020). During this Q-neuron-induced hypothermia and hypometabolism (QIH), neural activity was substantially reduced in the whole brain without any observed tissue damage following hibernation. This tool would be a fascinating approach to help us gain insights into mechanisms of latent period, plasticity changes, and their role in TLE development. More research is needed to delineate the stages of TLE so that we may be better able to evaluate and perhaps halt the progression of TLE from the perspective of pathological oscillations.

## Author Contributions

Both authors listed have made a substantial, direct, and intellectual contribution to the work, and approved it for publication.

## Conflict of Interest

The authors declare that the research was conducted in the absence of any commercial or financial relationships that could be construed as a potential conflict of interest.

## Publisher’s Note

All claims expressed in this article are solely those of the authors and do not necessarily represent those of their affiliated organizations, or those of the publisher, the editors and the reviewers. Any product that may be evaluated in this article, or claim that may be made by its manufacturer, is not guaranteed or endorsed by the publisher.
